# Prediction of immunogenicity for humanized and full human therapeutic antibodies

**DOI:** 10.1371/journal.pone.0238150

**Published:** 2020-08-31

**Authors:** Shide Liang, Chi Zhang

**Affiliations:** 1 Department of Research and Development, Bio-Thera Solutions, Guangzhou, P. R. China; 2 School of Biological Sciences, University of Nebraska, Lincoln, NE, United States of America; University of Lincoln, UNITED KINGDOM

## Abstract

Immunogenicity is an important concern for therapeutic antibodies during drug development. By analyzing co-crystal structures of idiotypic antibodies and their antibodies, we found that anti-idiotypic antibodies usually bind the Complementarity Determining Regions (CDR) of idiotypic antibodies. Sequence and structural features were identified for distinguishing immunogenic antibodies from non-immunogenic antibodies. For example, non-immunogenic antibodies have a significantly larger cavity volume at the CDR region and a more hydrophobic CDR-H3 loop than immunogenic antibodies. Antibodies containing no Gly at the turn of CDR-H2 loop are often immunogenic. We integrated these features together with a machine learning platform to Predict Immunogenicity for humanized and full human THerapeutic Antibodies (PITHA). This method achieved an accuracy of 83% in leave-one-out experiment for 29 therapeutic antibodies with available crystal structures. The accuracy decreased to 65% for 23 test antibodies with modeled structures, because their crystal structures were not available, and the prediction was made with modeled structures. The server of this method is accessible at http://mabmedicine.com/PITHA.

## Introduction

Since the first recombinant therapeutic protein, human insulin, was approved in 1982, more than 250 products have entered the marketplace with an estimated annual revenue of over 150 billion dollars. Therapeutic proteins including monoclonal antibodies, coagulation factors, replacement enzymes, fusion proteins, hormones, growth factors, and plasma proteins are now a fast-growing segment of the pharmaceutical industry. These approved therapeutic proteins are indicated for a wide variety of areas such as cancers, autoimmunity/inflammation, exposure to infectious agents, and genetic disorders [1, 2]. The rapid advances in biomedical science and technology make it possible to address the unmet needs with new therapeutic proteins.

In comparison with small molecules that bind in a deep pocket, biopharmaceuticals can bind the flat surface of a protein with high specificity to interfere *in vivo* processes and restore previously untreatable conditions. However, when therapeutic proteins are administrated to patients, unwanted immune responses, such as a generation of anti-drug antibody (ADA), can cause a wide range of problems including altered pharmacokinetics, loss of efficacy, and even life-threatening complications as reviewed in references [3–5]. The immunogenicity against therapeutic proteins can be generated in both T cell dependent and T cell independent pathways. Antibodies generated from T cell dependent pathway have a higher affinity than those generated from T cell independent activation and appear to play a critical role in the development of antibody responses to biologic therapeutics [6]. More specifically, T cells are activated by the recognition of linear antigenic peptides derived from the therapeutic proteins, called T cell epitope. The activated T cells then stimulate B cells to generate ADAs against the therapeutic protein.

Unlike molecules recognizing T cell epitopes, antibodies bind a conformational epitope on the protein surface, called B cell epitope. The existing tools predict the sequence-discontinuous B cell epitope based on the physiochemical properties of a protein structure and the performance is far from ideal with an accuracy slightly better than random [7–13]. Linear B cell epitope prediction algorithms were also developed even though 90% of B cell epitopes were considered conformational [14]. Nevertheless, experimental methods have been developed to identify B cell epitopes for therapeutic proteins using human anti-serum from previously treated patients [15] or structure-guided design via antibody-antigen co-crystals [16]. The epitope could be deleted while retaining the therapeutic function by sequential rounds of mutagenesis and testing [17]. For the mouse model, the deimmunized PE38, which is a 38-kDa portion of *Pseudomonas* exotoxin A, did not induce the formation of antibody in mice after being repeatedly applied by intravenous injection [18]. In addition, the immunogenicity could be minimized by controlling critical quality attributes of the therapeutic proteins [19]. More frequently, T cell epitopes were predicted and deleted for biotherapeutic deimmunization [20–22], partly due to the difficulty of direct prediction of B cell epitopes. So far computational prediction of T cell epitopes achieved significant progress [14]. Numerous prediction algorithms have been developed and an AUC (area under curve) value of 0.786 was obtained for a large test set by consensus approach [23].

Monoclonal antibodies contributed almost half of therapeutic proteins approved by the U.S. Food and Drug Administration (FDA) in the past several years [1]. Immunogenicity is an important concern for therapeutic antibodies during drug development and regulation. For example, bococizumab, a humanized monoclonal antibody being developed to reduce the levels of low-density lipoprotein cholesterol, was recently discontinued after phase III clinical trials on 4300 patients citing decreased treatment efficacy due to high immunogenicity incidence rates [24]. Since accurate prediction of B cell epitopes for general therapeutic proteins was an elusive task [14], we intended to develop an algorithm only for distinguishing immunogenic antibodies from non-immunogenic antibodies based on B cell epitope properties. The rich information of immunogenicity could be obtained on the FDA’s website for each therapeutic antibody.

Currently, few mouse antibodies are in clinical development stage for the therapeutic purpose due to immunogenicity. In fact, simple replacement of mouse immunoglobulin constant regions with human ones results in significant immunogenicity reduction for the chimeric antibodies. Humanization of variable fragment (Fv) results in a further decrease of immunogenicity [25]. Compared to humanized antibodies, however, full human antibodies selected from transgenic mice or phage display platforms [26], show almost no difference in immunogenicity in spite of the highest humanness score [27]. In recent years, humanized and full human antibodies constituted most of the approved therapeutic antibodies. We performed a statistical analysis of the sequence and structural properties at the CDR regions, where B cell epitopes could reside, for the two types of antibodies. Several features related to immunogenicity, such as cavity volume and hydrophobicity of CDR-H3 loop, were identified. We integrated all features together with a Support Vector Machine (SVM) learning algorithm to Predict Immunogenicity for humanized and full human THerapeutic Antibodies (PITHA).

## Results

### Irrelevance of humanness scores and immunogenicity

To predict their immunogenicity, it is important to identify therapeutic antibody sequence and structural features to distinguish immunogenic antibodies from non-immunogenic antibodies. We investigated two frequently used terms in antibody immunogenicity research: humanness score and T cell epitope prediction. The humanness score, i.e., the distance to the human consensus sequence, was defined in a different way from other methods [27, 28]. Specifically, we focused on the CDR regions to maximize the gap between the calculated values for different types of antibodies. Indeed, the humanness scores of full human antibodies are significantly higher than those of humanized antibodies at the CDR region (*p* value < 10^−15^) but the difference is minimal for the framework (Table 1). In addition, the ratio of immunogenic antibodies to non-immunogenic antibodies is similar for humanized antibodies (1.1) and full human antibodies (1.3), we infer that the immunogenicity of an antibody is not related to the humanness score of its CDR region. The immunogenicity of murine antibodies could be minimized by substituting the framework residues, not frequently observed in human antibodies, with common ones during humanization.

**Table 1:** Similar humanness scores for immunogenic and non-immunogenic therapeutic antibodies.

Antibodies	Type (number)	CDR humanness	CDR rare residues	Framework humanness	Framework rare residues
High immunogenicity	Full human (14)	-82 ± 19	4.0 ± 1.8	-21 ± 14	1.6 ± 1.5
	Humanized (14)	-172 ± 24	12.9 ± 3.1	-32 ± 18	2.1 ± 2.0
Low immunogenicity	Full human (11)	-77 ± 29	4.2 ± 2.1	-25 ± 34	1.5 ± 2.5
	Humanized (13)	-178 ± 38	14.5 ± 4.5	-36 ± 22	2.8 ± 2.0

The humanness score was calculated as a negative number close to zero for human-like sequences and large in magnitude for non-human-like sequences.

**Fig 1:**
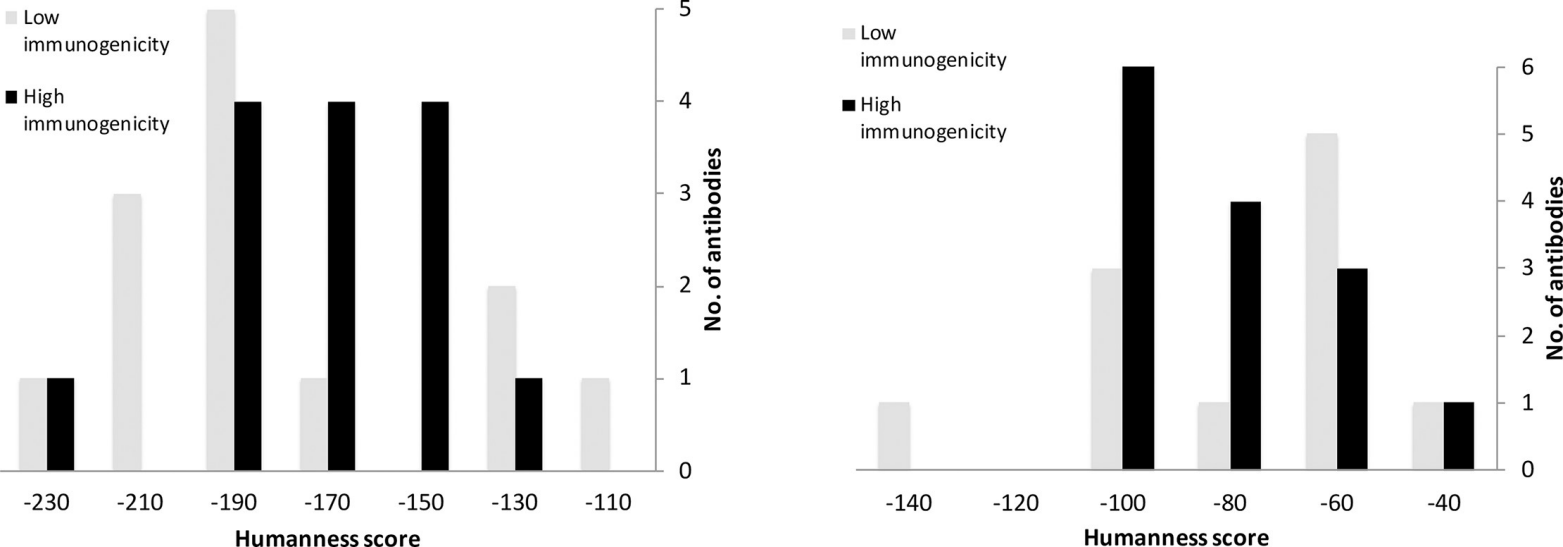
Distribution pattern of calculated humanness scores for CDR regions of immunogenic and non-immunogenic therapeutic antibodies. (a) Humanized antibodies. (b) Full human antibodies.

Unfortunately, when the 52 antibodies were divided into humanized antibodies and full human antibodies, the mean value of the calculated humanness scores of immunogenic antibodies was equivalent to that of non-immunogenic antibodies and the distribution ranges were similar within either group (Fig 1). That is, one could not predict the immunogenicity for therapeutic antibodies based on the humanness score.

### Irrelevance of T cell epitope prediction results and immunogenicity

T cell epitope is one of factors contributing to immune responses. The activation of helper T cell is essential for B cell proliferation, antibody class switching, and an increase in antibody production. Recognition of linear epitopes bound by MHC class II molecules on the surface of antigen presenting cells is a critical step for T cell activation. We predicted T cell epitopes for the Fv domains of immunogenic and non-immunogenic antibodies using the consensus method [23] and the online server (http://tools.iedb.org/mhcii/) in April 2018. On average, immunogenic antibodies do not contain more fragments as a good binder against the full reference set of MHC class II alleles than non-immunogenic antibodies for both CDR regions and the whole Fv domain (Table 2). The immunogenicity is not correlated with the affinity of the best binder among total fragment-allele pairs either. The results were essentially indistinguishable for the two types of antibodies, even when we changed the cut-off value for the predicted T cell epitopes. Therefore, the immunogenicity of therapeutic antibodies could not be inferred by T cell epitope prediction results.

**Table 2:** T cell epitope prediction results averaged for immunogenic and non-immunogenic antibodies.

Antibodies	Minimum rank^a^	Allele frequency^b^	Total good binders^c^	Good binders overlapping CDR
High Immunogenicity	0.12 ± 0.11%	18.5 ± 2.7	172 ± 49	98 ± 33
Low immunogenicity	0.08 ± 0.08%	18.7 ± 3.1	178 ± 45	103 ± 34

^a^The lowest rank (the best binder) calculated for a reference panel of 27 alleles against all fragments of the Fv domain of an antibody.

^b^The number of alleles if they predicted as a percentile rank < 3% against anyone of the Fv fragments.

^c^The number of total allele-fragment pairs with a percentile rank < 3%.

### Idiotype-anti-idiotype complex structures

**Fig 2:**
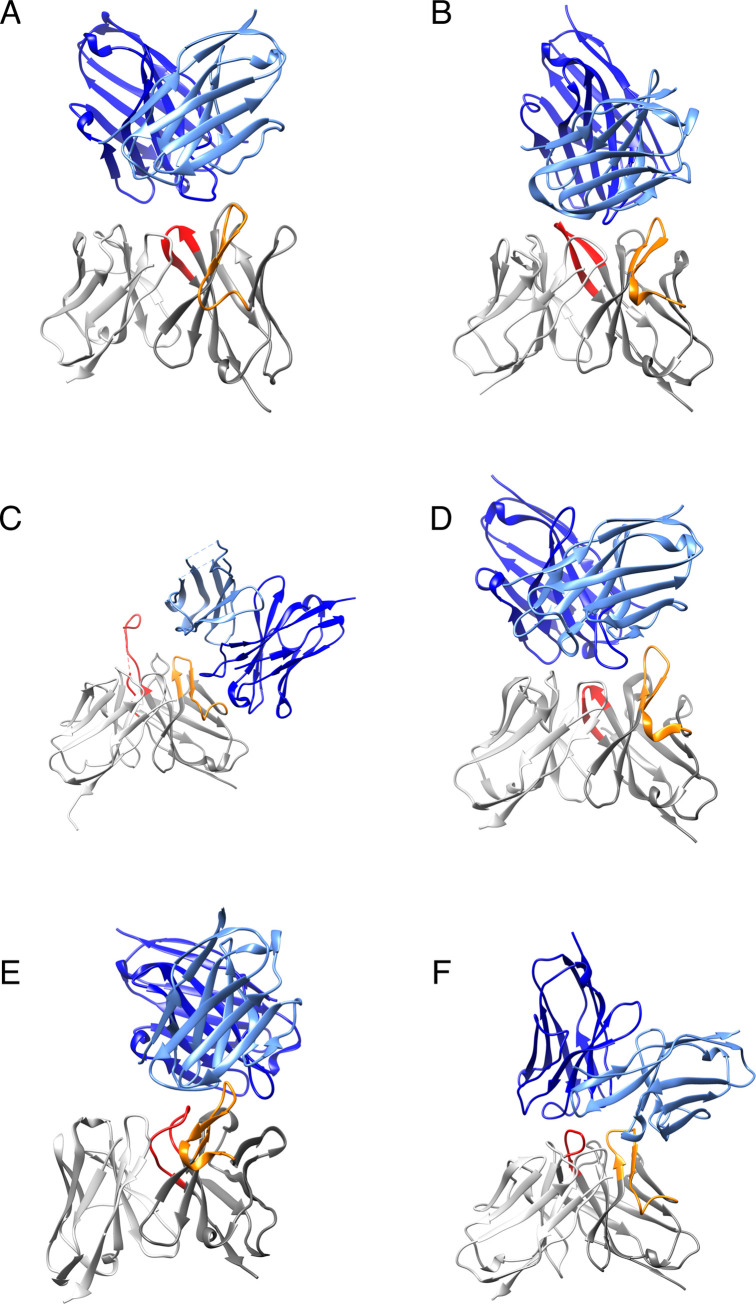
Crystal structures of idiotype-anti-idiotype Fv complex. Grey: idiotypic antibody; Orange: CDR-H2 of idiotypic antibody; Red: CDR-H3 of idiotypic antibody; Light-blue: light chain of anti-idiotypic antibody; Deep-blue: heavy chain of anti-idiotypic antibody. The PDB IDs are shown for complex structures—A. 1dvf, idiotopic antibody D1.3-anti-idiotopic antibody E5.2; B. 1iai, idiotopic antibody 730.1.4-anti-idiotopic antibody 409.5.3; C. 3bqu, idiotopic antibody 2F5-anti-idiotopic antibody 3H6; D. 1pg7, idiotopic antibody D3H44-anti-idiotopic antibody 6A6; E. 5jo4, idiotopic antibody D80-anti-idiotopic antibody G6; F. 5xaj, idiotopic antibody HM14c10-anti-idiotopic antibody E1.

The crystal structure of an idiotype-anti-idiotype complex precisely shows how the anti-idiotypic antibodies bind the idiotypic antibodies. We searched the PDB database with the key words “antibody” and “complex” and found 6 idiotype-anti-idiotype complexes from 1883 results in April 2018. As shown in Fig 2A–2F, the anti-idiotypic antibodies bind exclusively to the CDR regions of the idiotypic antibodies, especially, the CDR-H2 loop and the CDR-H3 loop. Only a few atoms at the framework of the idiotypic antibodies, i.e., B cell epitopes residing in the CDR regions, make direct contact with the anti-idiotypic antibody (Fig 2C). Motivated by the crystal structures of the 6 complexes, we focused on the CDR regions in the following studies in order to identify features that distinguish immunogenic antibodies from non-immunogenic antibodies.

### Cavity at CDR region

**Fig 3:**
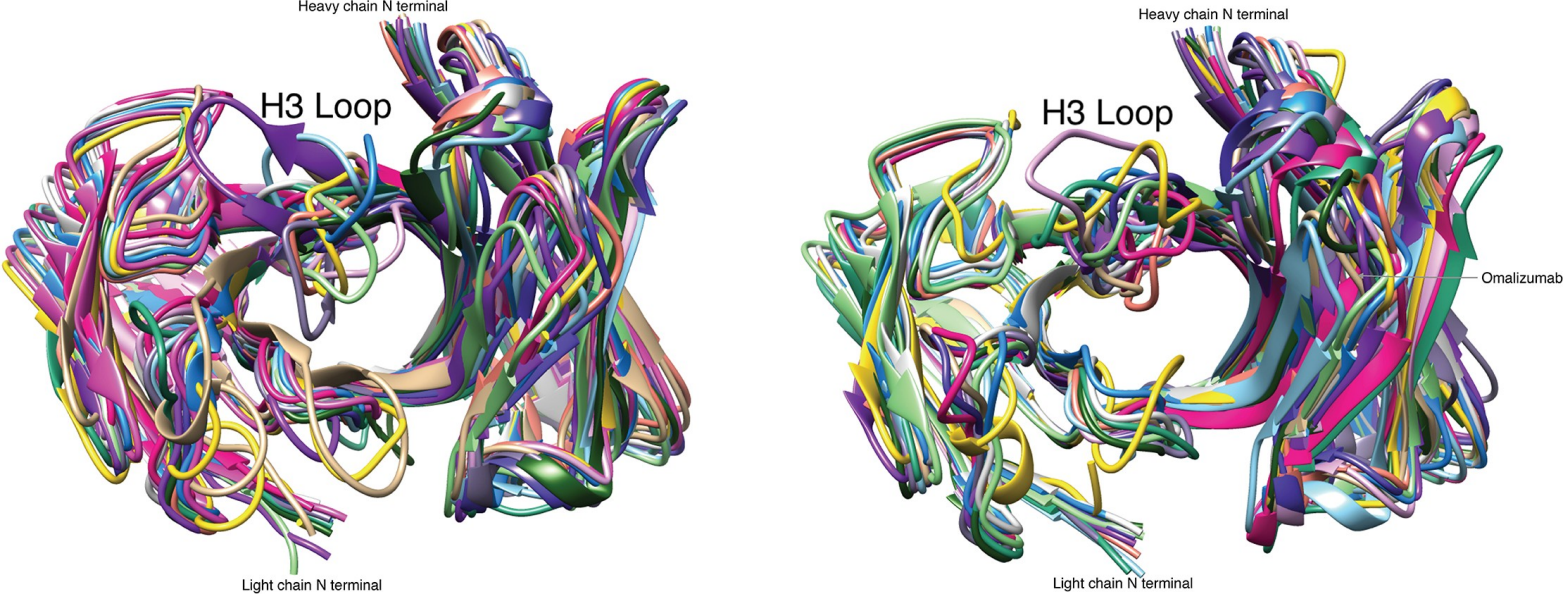
Comparison of backbone structures for immunogenic and non-immunogenic therapeutic antibodies. (a) 15 immunogenic antibodies. (b) 14 non-immunogenic antibodies. The crystal structures of all the antibodies were obtained from PDB and superimposed to that of omalizumab (tan in Fig 3B).

By visual analysis of crystal structures (Fig 3), we found that the central cavities between the heavy chains and the light chains of immunogenic antibodies were smaller than those of non-immunogenic antibodies. In addition, the length of CDR-H3 loops of the 15 immunogenic antibodies (9.7 residues in average) is slightly shorter than that of the 14 non-immunogenic antibodies (11.4). The CDR-H3 loops of immunogenic antibodies tend to form a curve to fill the cavity at the CDR region, providing a smooth surface at the CDR region of an idiotypic antibody that allows the binding of anti-idiotypic antibodies (Fig 3A and 3B). On the contrary, the relatively long and rigid CDR-H3 loops of non-immunogenic antibodies protrude directly into solvent, which makes it difficult for the anti-idiotypic antibodies to bind the CDR regions (Fig 3B).

For quantitative evaluation, we used the FPOCKET program [29] to identify the cavities at the CDR regions for all-atom antibody structure and calculate the total cavity volumes (Table 3). Consistent with visual analysis, the cavity volume of immunogenic antibodies (361 Å^3^ in average) was found to be smaller than that of non-immunogenic antibodies (798 Å^3^ in average). The difference is statistically significant according to Student’s *t*-test (*p* value <0.05). Due to the large size of CDR regions, one can conclude that anti-idiotypic antibodies are not likely to bind the surface patch with a deep cavity in CDR regions, which is, however, ideal for the binding of a small molecule on the other hand.

**Table 3:** Features distinguishing immunogenic antibodies from non-immunogenic antibodies.

Antibody (PDB code)	Cavity volume (Å^3^)	CDR-H3 Hydrophobic surface area (Å^2^)	Gly at CDR-H2 turn
High immunogenicity			
Adalimumab(4nyl)	0	340	1
Panitumumab(5sx4)	0	176	1
Ustekinumab(3hmw)	0	310	0
Avelumab(4nki)	518	285	2
Durvalumab(5x8m)	536	482	1
Necitumumab(6b3s)	138	327	1
Nivolumab(5wt9)	334	157	1
Ramucirumab(3s34)	338	114	0
Guselkumab(4m6n)	556	220	0
Certolizumab(5wuv)	1224	310	1
Daclizumab(3nfs)	0	112	1
Efalizumab(3eo9)	0	319	0
Natalizumab(4irz)	506	374	1
Atezolizumab(5x8l)	684	263	2
Obinutuzumab(3pp4)	576	304	2
Mean	361	273	
	Low immunogenicity		
Canakinumab(4g5z)	342	244	1
Ofatumumab(3giz)	1173	452	1
Ipilimumab(5tru)	0	274	1
Belimumab(5y9j)	1425	584	1
Bevacizumab(1bj1)	1045	556	1
Eculizumab(5i5k)	1077	549	2
Omalizumab(4x7s)	942	357	1
Palivizumab(2hwz)	825	318	0
Trastuzumab(6bhz)	731	256	1
Alemtuzumab(1bey)	0	228	1
Pembrolizumab(5ggs)	2081	410	2
Ibalizumab(3o2d)	1524	414	1
Pertuzumab(1s78)	0	251	2
Bezlotoxumab(4np4)	0	295	1
Mean	798	371	

Crystal structures were used for statistical analysis.

### Hydrophobicity of CDR-H3 loop

Hydrophobic interactions play a critical role for the tight binding of a protein complex. The hydrophobicity of CDR-H3 loop is estimated as the percentage of solvent accessible surface of all hydrophobic atoms in the total surface. However, no hydrophobicity difference was observed for the whole CDR region between immunogenic antibodies (58.5 ± 2.8%) and non-immunogenic antibodies (59.2 ± 2.4%). The percentage of hydrophobic surface area in the total surface area actually varies within a small range for protein stability reasons. Since hydrophobic interactions are favorable for protein association, theoretically, it is easy for the anti-idiotypic antibodies to bind the therapeutic antibodies with a hydrophobic CDR-H3 loop, which usually is located at the center of the binding site (Fig 2A–2F). To our surprise, the CDR-H3 loops of immunogenic antibodies have a significantly smaller hydrophobic surface area (*p* value <0.05) than that of non-immunogenic antibodies (Table 3). This is not caused by the slight difference of loop length. In fact, the ratio of hydrophobic surface to total surface is also smaller for the CDR-H3 loops of the 15 immunogenic antibodies (62.5 ± 10.2%) than that of the 14 non-immunogenic antibodies (67.4 ± 4.3%). We assume that the antigen receptors, which potentially bind the therapeutic antibody with a hydrophobic CDR-H3 loop, also bind similar self-antibodies. As a result, the immature B cells with the cross-reactive antigen receptors on the surface are eliminated or inactivated during the early development and the foreign antibodies with a hydrophobic CDR-H3 show low immunogenicity.

### Number of Gly at CDR-H2 turn

Besides the CDR-H3 loop, the CDR-H2 loop is frequently located at the center of the anti-idiotypic antibody epitopes on idiotypic antibodies (Fig 2A–2F). In general, the β turn of CDR-H2 loop (VH 52–56) is glycine rich for antibodies from various species for structural reasons but contains no glycine in 7 out of the 52 therapeutic antibodies. Interestingly, 6 of the 7 antibodies are immunogenic. Despite the small size of the analyzed data set, we inferred that antibodies without glycine at the CDR-H2 turns are immunogenic. Actually, one humanized antibody, huBrE-3, does not contain any CDR-H2 loop, and anti-drug antibodies were detectable in 1 out of 7 patient’s serum in its initial clinical evaluation [30].

### Prediction of immunogenicity

Support vector machine (SVM) learning technology was used to integrate the features discussed above for immunogenicity prediction. The method achieved an impressive accuracy of 83% for the 29 therapeutic antibodies when the two features, cavity volume at the CDR region and hydrophobicity of CDR-H3 loop, calculated from the crystal structures were used in the leave-one-out experiment. The accuracy, however, was decreased to 76% by combining the information of presence/absence glycine at CDR-H2 turns additionally due to over fit resulting from the small data set. Moreover, the SVM model trained with the two effective terms of the 29 antibodies shows no predictive ability (48% accuracy) for the 23 test antibodies, of which the crystal structures are unavailable, and the modeled structures have to be used for prediction.

We found that the cavity volume calculated by FPOCKET could be significantly affected by the coordinate errors for the modeled structures resulting in low prediction accuracy. To investigate the effect of inaccurate structures, we used ABodyBuilder [31] to predict protein structures for 5 immunogenic antibodies, Adalimumab, Panitumumab, Ustekinumab, Daclizumab, and Efalizumab (Table 3), of which no cavities at the CDR regions were identified in their crystal structures. Their observed structures were excluded from the template library for the prediction. Using the modeled structures, we found cavities at the CDR region for all of 5 antibodies with an average volume of 922 Å^3^, which makes them indistinguishable from non-immunogenic antibodies. On the other hand, the exposed hydrophobic surface area of CDR-H3 of the modeled structures is fairly consistent with that of the observed structures for the 5 antibodies despite a small increase in most cases (339, 310, 245, 168, and 398 Å^2^ versus 340, 176, 310, 112, and 319 Å^2^, respectively). We thus utilized another set of features, hydrophobicity of CDR-H3 and the information of the presence/absence of glycine at CDR-H2 turn, for SVM classification and achieved an accuracy of 79% in leave-one-out experiment for the 29 antibodies calculated with crystal structures. When the trained SVM model was used for the modeled structures of 23 test antibodies (Table 4), a meaningful accuracy of 65% was obtained compared to no predictive ability of the above-mentioned SVM model, for which the training terms contain the cavity volume at the CDR region. Therefore, dependent on availability of the crystal structure, different SVM models trained with appropriate features should be used to maximize the prediction accuracy.

**Table 4:** Prediction results for the modeled structures of therapeutic antibodies.

Antibody	CDR-H3 Hydrophobic surface area (Å^2^)	Gly at CDR-H2 turn	Prediction results^a^
High immunogenicity			
Golimumab	589	1	0
Alirocumab	165	3	1
Dupilumab	653	3	0
Olaratumab	581	1	0
Sarilumab	123	1	1
Elotuzumab	239	0	1
Ixekizumab	354	1	1
Mepolizumab	289	2	1
Reslizumab	262	1	0
Vedolizumab	463	0	1
Inotuzumab	304	1	1
Benralizumab	482	1	0
Tildrakizumab	126	1	1
Mean	356		
Low immunogenicity			
Denosumab	456	3	0
Daratumumab	715	4	0
Evolocumab	215	1	0
Raxibacumab	334	3	1
Secukinumab	874	1	1
Burosumab	219	1	0
Tocilizumab	422	1	0
Gemtuzumab	178	1	1
Idarucizumab	510	2	0
Ocrelizumab	399	2	0
Mean	432		

^a^Predicted immunogenic and non-immunogenic antibodies were indicated by “1” and “0”, respectively.

As shown in Tables 3 and 4, the exposed hydrophobic surface areas of CDR-H3 of immunogenic antibodies are smaller than that of non-immunogenic antibodies for either crystal structures or modeled structures. However, the surface area of CDR-H3 could be systematically overestimated for the structures modeled by ABodyBuilder. Since the crystal structures usually are unavailable for the predicted antibodies, it is reasonable to use the modeled structures for SVM model training and prediction consistently. When hydrophobicity of CDR-H3 loops calculated from the modeled structures and the information of the presence/absence of Gly at CDR-H2 turns were used for SVM classification, we achieved an accuracy of 78% in leave-one-out experiment for the 23 test antibodies, which do not have crystal structures. We then used the trained SVM model to predict the immunogenicity for 11 therapeutic antibodies approved after April 2018 including cemiplimab, emapalumab, erenumab, fremanezumab, galcanezumab, lanadelumab, mogamulizumab, polatuzumab, ravulizumab, risankizumab, and romosozumab. The prediction was made with modeled structures and the result was correct for 7 out of the 11 antibodies. Similarly, we successfully predicted the high immunogenicity of bococizumab, which was discontinued after phase 3 clinical trial [24].

## Discussion

The observed immunogenicity of therapeutic proteins is highly dependent on several factors including assay methodology, underling disease, concomitant medications. During data collection, if possible, we used the data generated at the same conditions to distinguish immunogenic antibodies from non-immunogenic antibodies. For example, when the test results of multiple doses were available, the rate of antibody development was considered for patients receiving 10 mg/kg or similar dose.

In this study, we considered B cell epitopes only at the CDR regions of idiotypic antibodies. However, for some cases, one allelic form of a therapeutic antibody can be immunogenic in patients of other allotypes and provoke antibody responses as a result of allo-immunization due to polymorphisms of the gene encoding the constant domains of human heavy chains [32]. Nevertheless, all six anti-idiotypic antibodies bind the CDR regions of the idiotypic antibodies, respectively, based on the available crystal structures (Fig 2). Recently, the crystal structure of the therapeutic antibody natalizumab in complex with anti-idiotyic antibody NAA32 was released (PDB ID: 6fg1). As expected, NAA32 also binds the CDR region of natalizumab.

In an early study, T cell epitopes were found only in CDR-sequence containing regions for a set of eight humanized antibodies [33]. A human helper T cell assay was also developed for assessing antibody immunogenicity *in vitro* [34]. The positive rate of four immunogenic antibodies was higher than that of two non-immunogenic antibodies for the evaluated blood samples. In addition, several recombinant proteins other than antibodies were deimmunized by removing T cell epitopes and assessed *in vivo* by anti-drug antibody titers [20, 35, 36]. Here, we found no correlation between immunogenicity and T cell epitope prediction results for the 52 humanized and full human antibodies. Since human immune system is well regulated and adaptive, the results of *in vitro* experiments may not correlate with the clinical outcomes. For example, the presence of regulatory T cell epitopes in antibody Fc region could lead to a suppression of effector cytokine secretion and reduced proliferation of effector T cells *in vivo* [37]. The immune responses could be suppressed in spite of the occurrence of other T cell epitopes.

As revealed by clinical trials in the past several years, the immunogenicity is nearly identical for humanized and full human antibodies regardless of the significantly different humanness scores calculated for the two groups of antibodies (Table 1). Humanizing an antibody may be sufficient to eliminate immunogenicity issues to the same extent as using full human antibodies [27]. The algorithm developed in this study could provide extra benefit to select candidate antibodies with low immunogenicity for clinical trials, especially, when idiotypic antibodies’ crystal structures are available. Although the accuracy is far from ideal, a humanized antibody, which is predicted as low immunogenicity by computational tools, could be a better choice for clinical development than the full human antibody generated from the expensive platform of transgenic mice.

## Materials and methods

### Sequences and structures of antibody variable regions

Sequences for the approved therapeutic antibodies were downloaded from the United States patent applications (patft.uspto.gov) and the KEGG drug database (https://www.genome.jp/kegg/drug). We collected 52 humanized and full human antibodies (Tables 3 and 4) approved by the FDA as of April 2018 excluding bispecific antibodies. Lucentis was also excluded for having a similar chemical origin to another approved antibody Avastin. Only the Fv domains were considered since the other parts of the collected antibodies are innate and essentially non-immunogenic. A total of 52 antibodies were used when sequence-based features were investigated. Kabat numbering, a scheme for the numbering of amino acid (AA) residues in antibodies, were used to assign numbers to the protein sequences of heavy chains (VH) and light chains (VL) with the software tool, ANARCI [38]. An expanded definition was used for the six loops at the CDR region in comparison with the classical Kabat, including the additional VH positions 26–30 for CDRH1 and 49 for CDR-H2.

Crystal structures were available for 29 out of the 52 antibodies in the Protein Data Bank (PDB, https://www.rcsb.org). Unless specifically indicated, we performed statistical analysis of structural features for the 29 proteins (Table 3). We also built structural models for the other 23 antibodies (Table 4) with the online server ABodyBuilder [31] to test the structure-based immunogenicity prediction algorithm developed in this study. Molecular graphics and analyses were performed with UCSF CHIMERA [39] for the protein structures.

### Immunogenic and non-immunogenic antibodies

The level of reported immunogenicity was obtained in the prescribing information for each therapeutic antibody at the FDA website (https://www.accessdata.fda.gov/scripts/cder/daf). Antibodies were classified as having low immunogenicity or being non-immunogenic when treatment-emergent anti-antibody response (AAR) was reported in less than 2% of patients. Otherwise, the antibodies were considered having high immunogenicity or being immunogenic. We called AAR detectable if patients in studies were tested at multiple time points and the AAR was detected at least once during treatment. If AAR data with and without concomitant immunosuppression were concurrently reported, results from patients not taking immunosuppressants were used.

We adopted this classification in order to have a similar number of immunogenic and non-immunogenic antibodies for the convenience of statistical analysis. As a result, 28 out of the 52 antibodies are considered immunogenic and the other 24 are non-immunogenic (Tables 3 and 4). Out of 29 antibodies with available crystal structures, 15 ones are immunogenic and the other 14 are non-immunogenic (Table 3).

### Definition of features for the prediction model

Residue humanness score is defined similarly to residue conservation score measured by the self-substitution score from the sequence profile as in the previous study [7]. Sequence profiles were obtained by three rounds of PSI-BLAST [40] searches against human sequences with the BLOSUM62 substitution matrix. The humanness score at position *i* is defined as

$$S_{human}=\left\{ \begin{array}{l} M_{ir}-B_{rr},\;if\;M_{ir}<B_{rr}\\ 0,\;if\;M_{ir}\geq B_{rr} \end{array}\right.$$

where *M*_ir_ is the self-substitution score in the position-specific substitution matrix generated from PSIBLAST for the residue type *r* at sequence position *i*, and *B*_rr_ is the diagonal element of BLOSUM62 for residue type *r*. We define *r* as a rare residue in case *S*_human_ is less than -6. The sum of humanness scores was calculated for residues at the CDR region and framework, respectively.

To calculate the cavity volume at the CDR region, the FPOCKET [29] program was used to identify all pockets in the Fv domain with default parameters. The program was originally developed to identify cavities at the protein surface likely to bind small compounds. We considered a pocket located at the CDR region if two third of the surrounding atoms of this pocket are of CDR residues. The sum of cavity volumes is calculated in case multiple pockets are identified at the CDR region.

The hydrophobicity of CDR-H3 loop is defined as the sum of solvent accessible surface of all hydrophobic atoms thereof. The carbon, sulfur, and nitrogen atoms (excluding the non-protonated nitrogen atom of histidine side chain) are considered hydrophobic atoms. Hydrogen atoms, which were missing in the crystal structures, were added with the REDUCE program [41]. The solvent probe was set to 1.4 Å. The atomic radii of carbon, nitrogen, oxygen, sulfur, and polar hydrogen were set to 1.8, 1.65, 1.4, 1.85, and 1.0 Å, respectively, and non-polar hydrogen atoms were ignored.

### Training and prediction procedure

To predict the immunogenicity of a therapeutic antibody, we extracted three types of features: cavity volume at the CDR region, hydrophobicity of CDR-H3 loop, and the information of presence/absence of Gly (1 or 0) at CDR-H2 turn. The library for support vector machines, LIBSVM-3.22, was downloaded from http://www.csie.ntu.edu.tw/~cjlin/libsvm and used for combining features and machine learning classification. The optimal parameters for model training were derived by the recommended method through cross-validation. The prediction accuracy is defined as the number of correctly predicted immunogenic and non-immunogenic antibodies divided by the number of total predictions.
